# Gravity-driven microfluidic synthesis of lipid nanoparticles via vibrating sharp-tip mixing

**DOI:** 10.1088/1758-5090/ae94b4

**Published:** 2026-08-17

**Authors:** Toktam Godary, Zhengru Liu, Peng Li

**Affiliations:** 1 C. Eugene Bennett Department of Chemistry, West Virginia University, Morgantown, WV, United States of America; 2 Department of Chemistry, Lehigh University, Bethlehem, PA, United States of America

**Keywords:** gravity-driven flow, microfluidics, lipid nanoparticles, nanoparticle synthesis

## Abstract

Lipid nanoparticles (LNPs) are widely used in nanomedicine, yet their energy-efficient manufacture with precise control over particle uniformity remains challenging. Here we report a gravity-driven microfluidic platform that integrates acoustically actuated sharp-tip mixing to enable high-throughput LNPs synthesis without the need for active pumps. By optimizing microchannel geometry, actuation conditions, and the spatial positioning of the mixing vortex, we achieve stable gravity-driven flow and complete mixing at total flow rates (TFRs) of up to 5 ml min^−1^. The optimized system produces LNPs with tunable sizes below 60 nm, polydispersity <0.3, and high production yields. We further show that nanoparticle uniformity is governed not only by mixing strength but also by where mixing and nucleation occur within the microchannel. These findings establish gravity-driven sharp-tip microfluidics as a scalable and energy-efficient strategy for controlled nanoparticle manufacturing, offering a practical route toward greener nanomedicine production.

## 1. Introduction

Lipid nanoparticles (LNPs) have attracted substantial attention over the past two decades due to their remarkable potential in nanomedicine. This interest was initially sparked by the first clinical approval of Doxil in 1995 and has since been sustained by the continued clinical success of LNPs in delivering both hydrophilic and hydrophobic therapeutics [1, 2]. Their excellent entrapment efficiency, combined with their biodegradability and biocompatibility properties, further enhances the appeal of LNPs as safe and effective carrier—not only for conventional drug molecules but also for nucleic acids such as mRNA and siRNA [3].

Due to this growing interest in LNPs within the biomedical industry, there is a continuous demand for the development and optimization of efficient synthesis methods. In this regard, various techniques have been employed to produce LNPs, including high-pressure homogenization, microemulsion, sonication, phase inversion, membrane contactor methods, and others [4]. Among all the methods, nanoprecipitation is considered as one of the most advantageous strategies for large-scale manufacturing, primarily due to its simplicity, the absence of any need for heating, as well as its cost friendly, versatility and ease of implementation [5].

In this method, ethanolic lipid solution, produced from dissolving lipid molecules in ethanol, mixes rapidly with an aqueous solution. The rapid dilution of the ethanolic lipid solution with an aqueous buffer initiates lipid self-assembly, which in turn triggers particle nucleation and subsequent growth [6]. The conventional bulk nanoprecipitation method for synthesizing LNPs has limited control over the synthesis process, often resulting in batch-to-batch variations in physicochemical properties of the nanoparticles [7]. In contrast, microfluidic synthesis method not only overcomes the limitations of bulk synthesis such as poor reproducibility and lack of product uniformity but also enables precise control over the synthesis process and fluid mixing, along with efficient mass transport and ease of automation [8].

Typically, microfluidic LNP synthesis employs various types of active pumps. According to the principles of hydraulic resistance, the pressure required to drive flow scales inversely with the fourth power of the channel diameter. Generating high flow rates in micro-scale channels requires substantial pressure, which makes pumps highly energy-consuming, costly, and bulky for high throughput synthesis [9]. In microscale channels, the energy required for pumping increases with the decrease of channel cross section and increase of channel length. To achieve high throughput synthesis, high-pressure pumps are inevitably needed.

Flow in microchannels can also be passively driven without syringe pumps by employing forces such as gravity and capillary action [10]. While capillary flow is generally limited to small channel dimensions and very low flow rates (ranging from nl min^−1^ to *μ*l min^−1^), gravity-driven flow presents an attractive alternative, offering high flow rates (ranging from *μ*l min^−1^ to ml min^−1^) with the lowest cost and energy by harnessing gravitational force [11, 12]. By adjusting the height difference, the flow within the microchannel can be easily tuned to achieve the desired flow rate and flow rate ratios (FRR). In addition to simplicity and energy efficiency, studies demonstrated that gravity-driven flow provides enhanced flow stability and more precise handling of streams compared to syringe pumps [13, 14].

Owing to the unique advantages of employing gravity-driven flow in microfluidics, this method has been adopted for many microfluidic applications including particle/cell separation, sorting, point-of-care diagnostics, cell culturing, and droplet generation or manipulation [15–22]. Despite the wide adoption by many applications, gravity-driven flow has rarely been used in nanoparticle synthesis. In a particular study by Minetti *et al*, gravity-driven flow was employed to synthesize curcumin-loaded albumin nanoparticles via hydrodynamic focusing microfluidic techniques based on molecular diffusion [23]. However, the flow rate of the synthesis is limited to ∼300 *μ*l min^−1^ with a height difference of 80 cm. Achieving low polydispersity in nanoparticle synthesis requires efficient mixing and precise control of flow profiles, which typically necessitates the use of small microchannels. However, the reduced dimensions of these channels substantially limit the flow rates attainable under gravity-driven conditions. As a result, to date, there have been no reports of gravity-driven nanoparticle synthesis operating at flow rates on the order of ml min^−1^.

Although gravity-driven flow is well suited for larger channel dimensions, which is particularly beneficial for mass-scale production, this approach has not yet found a way into industry. There are several limitations when utilizing gravity flow including flow rate consistency, potential disturbances due to air bubbles and pressure fluctuation, and the variations in viscosity for different LNP recipes. Additionally, there are challenges associated with mixing efficiency in microfluidic systems. As channel dimensions and flow rates increase, the mixing becomes insufficient, leading to incomplete mixing and the formation of polydisperse nanoparticles. Passive mixing strategies, which rely on microchannel geometry and internal structures to enhance mixing, are effective predominantly at small scales. In contrast, active mixing strategies use externally applied forces such as acoustic, electric, or magnetic fields to achieve enhanced mixing performance. While these approaches can overcome the spatial and flow rate constraints of passive systems, they do so at the expense of significantly increased energy consumption. This elevated energy demand poses a critical barrier to their feasibility in industrial-scale synthesis, where process efficiency and energy conservation are paramount. In this regard, there is a growing need for a gravity-driven microfluidic approach that is not confined to small channel dimensions yet capable of achieving efficient and complete mixing at high flow rates with excellent tolerance on potential flow fluctuations.

Here, we introduce a gravity pump-based approach that challenges this conventional limitation, achieving high-throughput nanoparticle synthesis at ml min^−1^ flow rates without compromising size uniformity. This platform leverages a sharp-tip mixing method that can achieve efficient fluid mixing in mm-sized channels, enabling precise control over size and size distributions of LNPs [24, 25]. Compared with previous studies, the present work optimized the main channel dimension and the inlet geometry where two streams first meet. As a result, the maximum allowed flow rates increased from 750 *µ*l min^−1^ and 1500 *µ*l min^−1^–5000 *µ*l min^−1^ while maintaining efficient mixing and LNP production with a polydispersity <0.3, significantly exceeding the flow rate limit of existing gravity flow-based nanoparticle synthesis devices.

## 2. Materials and methods

### 2.1. Materials

Water was purified using a purification system from Merck Millipore in Bedford, MA, USA. Isopropanol and Ethanol were bought from Fisher Scientific in Pittsburgh, PA, USA. The Norland Optical Adhesive was purchased from Norland Products in Jamesburg, New Jersey, USA. Epoxy glue (5 min epoxy, Devcon) was purchased from ITW in Glenview, IL, USA and the glass glue was bought from Loctite in Rocky Hill, CT, USA. Conjured rigid 3D Printing resin was purchased from Chitu systems from Grace Place, CA, USA. Fluorescein sodium salt and sodium chloride were bought from Sigma − Aldrich in St. Louis, MO, USA. Phosphatidylcholine (POPC) was purchased from Avanti Lipids in Birmingham, AL, USA. Polytetrafluoroethylene extruded regular wall tubing with 0.8 mm inner diameter and 1.5 mm outer diameter was bought from Zeus Inc. In Orangeburg, SC, USA. Precision collection capillary tubes with 0.4 mm inner diameter and 75 mm length by Drummond Scientific in Broomall, PA, USA. Ultra-Ever Dry superhydrophobic top and bottom coating was purchased from Ultratech in Northeast, OH, USA. 1,2-Dioleoyl-3-trimethylammonium-propane (DOTAP) chloride, 1,2-Distearoyl-sn-glycero-3-phosphorylcholine (DSPC), and 1,2-Dimyristoyl-rac-glycero-3-methoxypolyethylene glycol-2000 (DMG-PEG 2000) were purchased from MedChemExpress in Monmouth Junction, NJ, USA. Cholesterol was purchased from Thermo Fisher Scientific in Ward Hill, MA, USA. 5’ 6-Carboxyfluorescein-labeled 20-mer ssDNA (5’-/6-FAM/TAA TAC GAC TCA CAT TAG GG-3’) was synthesized by Integrated DNA Technologies Inc. in Coralville, IA, USA. 2-[4-(2,4,4-trimethylpentan-2-yl)phenoxy]ethanol (Triton X100) was purchased from Sigma − Aldrich in St. Louis, MO, USA. Pierce™ Protein Concentrators PES (100 K MWCO, 2–6 ml) was purchased from Thermo Fisher Scientific in Rockford, IL, USA.

### 2.2. Instrumentation

Fluid mixing within the microchannel was observed using an Olympus IX-73 inverted fluorescence microscope. Characterization of the synthesized LNPs was performed using a NanoSight NS3000 and a ZetaSizer Pro, both from Malvern Panalytical. To fabricate sharp-tip glass capillaries, a PUL-100 microelectrode puller from World Precision Instruments was utilized. For generating acoustic streaming within the microchannel, a Tektronix arbitrary function generator paired with a Krohn-Hite 7500 wideband power amplifier was employed. An Avanti J–E Centrifuge (Beckman Coulter Life Sciences, Indianapolis, IN, USA) was used for sample centrifugation. A Horiba Fluorolog Fluorometer (HORIBA Instruments Incorporated, Irvine, CA, USA) was applied for fluorescence measurement.

### 2.3. Microfluidic device fabrication

Like our previous works, the microfluidic devices were fabricated with specific modifications on side channel for future optimization. Designs were created in SolidWorks and printed using a Sonic 8Ks 3D printer with conjured rigid transparent resin. The main microchannel was kept at a fixed length of 2800 *μ*m, height of 500 *μ*m, and width of 750 *μ*m. After printing, devices were rinsed with isopropanol, submerged in water, and cured under 405 nm UV light for three minutes. For improved structural integrity and microscopic visualization, each device was bonded to a glass slide using epoxy glue. A side channel was incorporated to connect the sharp-tip glass capillary to the main channel and coated with super hydrophobic solutions to prevent fluid leakage. The hydrophobic coating was applied in two steps. The base layer is applied first using a micropipette, followed by the top layer. Each layer is allowed to dry for one hour in a fume hood. Devices were then left to dry overnight before use.

**Figure 1.**
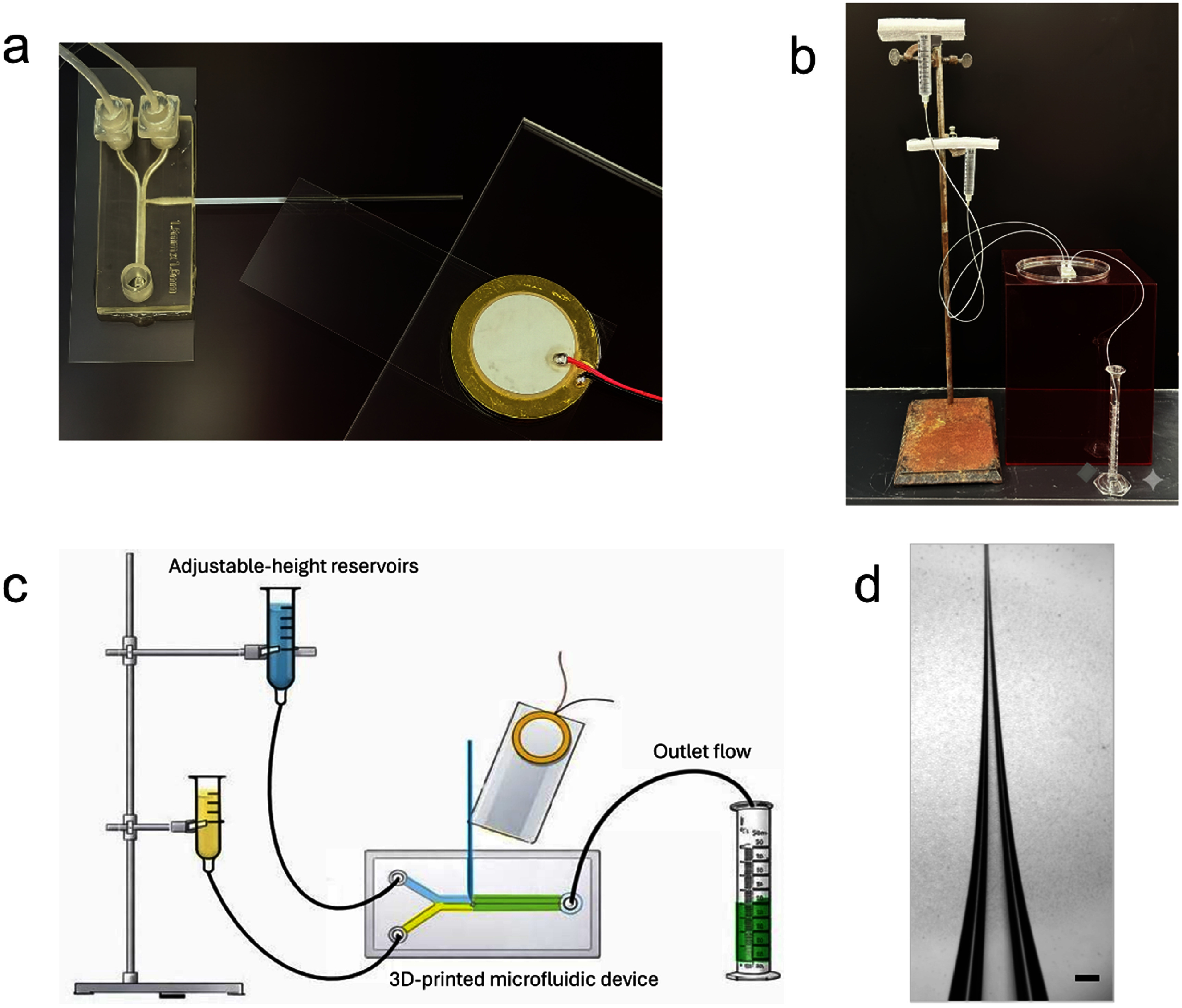
(a) Assembled sharp-tip glass capillary integrated with the 3D-printed microfluidic device. (b) Gravity-driven flow setup with two reservoirs positioned at calculated heights to achieve the desired TFRs and FRR, feeding into a dual-inlet microfluidic device. (c) Schematic of the gravity-driven setup, and (d) microscope image of a short graduated taper sharp-tip used in sharp-tip mixer device. The scale bar is 200 *µ*m.

The sharp-tip mixing device was fabricated using standard components: a piezoelectric transducer, glass capillary tube, and microscope slide. The transducer was first attached to the slide with epoxy glue. Sharp-tip capillaries were then fabricated using a PUL-1000 microelectrode puller under two different factory set pulling programs, each designed to generate capillaries with different taper geometries. Various taper geometries of sharp-tips were used to assess the stability of the capillary under gravity-driven system. Each capillary was glued at a 30° angle to the corner of the slide and coated up to its tip with a two-step superhydrophobic coating. Finally, the sharp-tip glass capillary was inserted into the side channel of the 3D-printed microfluidic device under a bright-field microscope. The device was ready for operation once the capillary was positioned such that its tip extended halfway into the main channel. The completed setup is presented in figure 1(a). The device runs stable across each lab section (typically 1–2 h) and is reusable across different days. Once assembled, the sharp-tip capillary and 3D printed channel were taped together to ensure reproducibility across different days of experiments.

### 2.4. Gravity-driven flow setup

The gravity-driven flow system employs two separate reservoirs: one filled with ethanol and the other with an aqueous solution. Both reservoirs have identical geometry, each being a 10 ml graduated container of the same shape and size. Each reservoir is positioned at an adjustable height to generate hydrostatic pressure that drives fluid flow through the microfluidic device. To accurately control and predict flow rates, a calibration step was performed for each fluid individually. In this step, a reservoir was connected to a single-inlet 3D-printed microfluidic channel, while the outlet tubing directed the fluid into a graduated cylinder. By measuring the volume of fluid collected over a fixed period of one minute, the flow rate corresponding to each reservoir height was determined. Due to the difference in viscosity between ethanol and the aqueous solution, each solvent experienced different flow resistance within the system. In this regard, the calibration was repeated at multiple heights for each fluid to establish precise height-to-flow rate relationships. It should be noted that each reservoir was filled to 10 ml prior to the start of every calibration experiment and refilled back to 10 ml after each run to ensure that every calibration test began with the same initial volume.

Once the flow rate profiles for each fluid were characterized, both reservoirs were set at specific heights calculated to achieve the desired TFR and FRR. The final gravity-driven flow setup is shown in figure 1(b), where each reservoir is connected to the 3D-printed microfluidic device through its own inlet. The outlet flow was collected to analyze the resulting fluid and validate the experimental results. A schematic representation of the complete gravity-driven flow setup is shown in figure 1(c).

It should also be noted that a short, graduated taper glass capillary was used for sharp-tip mixing device. As shown in figure 1(d), this tip geometry provided sufficient mechanical stability under gravity-driven flow conditions and showed reliably handled without breakage during operation. In contrast, longer taper sharp-tip capillaries were found to be mechanically unstable under the same conditions, frequently leading to tip breakage. Therefore, the short, graduated taper geometry was selected for all gravity-driven mixing experiments.

### 2.5. Mixing characterization

Following protocols from previous studies [25, 26], each sharp-tip capillary system was subjected to detailed mixing characterization to evaluate its performance. In brief, under Olympus IX-73 inverted fluorescence microscope, ethanol and fluorescein solutions flow into the microfluidic device from reservoir positioned at calculated height to achieve specific TFRs and FRRs. Next, acoustic streaming was induced by connecting the piezoelectric transducer to a signal generator. With a constant amplitude, the frequency was incrementally swept from 90 to 100 kHz, and fluorescence images were acquired at each step. Mixing index values were calculated using ImageJ by dividing the mean fluorescence intensity by the standard deviation; a value below 0.1 indicated uniform mixing [26]. The optimal frequency was identified based on the lowest mixing index, and amplitude was subsequently optimized by sweeping from 1 to 10 *V*_pp_ at that frequency. The combination of optimal frequency and amplitude was used to synthesize LNPs.

### 2.6. Nanoparticles synthesis and characterization

Synthesizing POPC LNPs was initiated by preparing two solutions: an ethanolic lipid solution and an aqueous saline solution. To prepare the lipid phase, 100 mg of POPC lipid powder was dissolved in 100 ml of ethanol to create an ethanolic lipid solution with 1 mg ml^−1^ concentration. The aqueous phase was prepared by dissolving 0.4 g of sodium chloride in 45 ml of Millipore-purified water. For nanoparticle synthesis, 10 ml of the ethanolic lipid solution and 10 ml of saline solution were loaded into separate reservoirs, which are connected by tubing to the respective inlets of the sharp-tip microfluidic mixing device. Then, the reservoirs were elevated to specific heights to establish gravity-driven flow, targeting TFRs of 1, 3 and 5 ml min^−1^. Based on previous findings indicating that a FRR of 1:3 results in optimal LNP production [24], this ratio was maintained constant throughout all experiments.

Once the setup was complete, the device was activated under its optimal acoustic mixing conditions, and the synthesized LNP solution was collected into microcentrifuge tubes. To ensure consistency and avoid cross-contamination, the device was thoroughly flushed with saline between each run. A minimum of 6 replicates were synthesized under each condition for statistical reliability. All collected samples were stored at −20 °C until characterization.

For characterization, nanoparticle’s size, size distribution and concentration of the POPC LNPs were measured using a Zetasizer Pro and a NanoSight NS300 nanoparticle tracking system (NTA). Due to the sensitivity of the NTA method, samples were diluted 400× based on the original concentration to achieve optimal particle visibility. Each sample was analyzed under dynamic conditions for 30 s, with three replicate measurements at a constant temperature of 25 °C and a syringe pump speed of 100.

### 2.7. DNA encapsulation

For DNA encapsulation, DOTAP was applied as a cationic lipid, DSPC and cholesterol were used as helper lipids, and DMG-PEG 2000 was used as a PEGylated lipid. The lipids were dissolved in ethanol at molar ratios of 50: 10: 38.5: 1.5 for DOTAP, DSPC, cholesterol, and DMG-PEG 2000, and the final concentration of lipid was 2 mg ml^−1^ in the ethanol solution. The aqueous DNA solution was prepared by diluting the 20-mer DNA standard to 1 µM (6.72 *µ*g ml) with a 25 mM ammonium acetate buffer.

The same gravity-flow set-up used for the synthesis of POPC nanoparticles was applied for DNA encapsulation with lipids. First, 5 ml of lipids solution and 5 ml of DNA solution were loaded into separated reservoirs. The heights of reservoirs were calibrated to realize a TFR of 1 ml min^−1^ and a FRR of 3:1 (0.75 ml min^−1^ for DNA solution and 0.25 ml min^−1^ for lipids solution). Then, the gravity-flow based DNA encapsulation in the microfluidics was processed under the optimal acoustic mixing conditions, and synthesized LNP loaded with DNA (DNA-LNP) were collected. Three replicates were collected in each synthesis and 0.5 ml of DNA-LNP solution was collected for each replicate. The DNA-LNP solution was then transferred to the ultrafiltration centrifugal concentrator (100 K MWCO), then centrifuged at 5000 g for 0.5 h to remove ethanol and unencapsulated DNA. After centrifugation, the concentrated DNA-LNPs were washed with deionized water and centrifuged at 5000 g for another 0.5 h. Finally, the DNA-LNP remained in the concentrator was resuspend in 0.4 ml of deionized water, collected, and diluted to 1 ml, which was stored at 4 ^o^C.

To evaluate the DNA encapsulation efficiency, the DNA-LNPs were broken to release the DNA loaded by adding 1 ml of 1% Triton X100 and then kept in darkness for 5 min. The fluorescence intensity of the DNA loaded in the DNA-LNP was then measured with a fluorometer. The amount of DNA loaded was quantified by the 6-FAM labeled DNA standards (diluted with 1% of Triton X100). The encapsulation efficiency is calculated by:

Encapsulation efficiency = (Mass of DNA loaded in the LNPs)/(Total mass DNA in the collected sample)

## 3. Results and discussion

### 3.1. Calibration of flow rates for the gravity-driven flow

**Figure 2.**
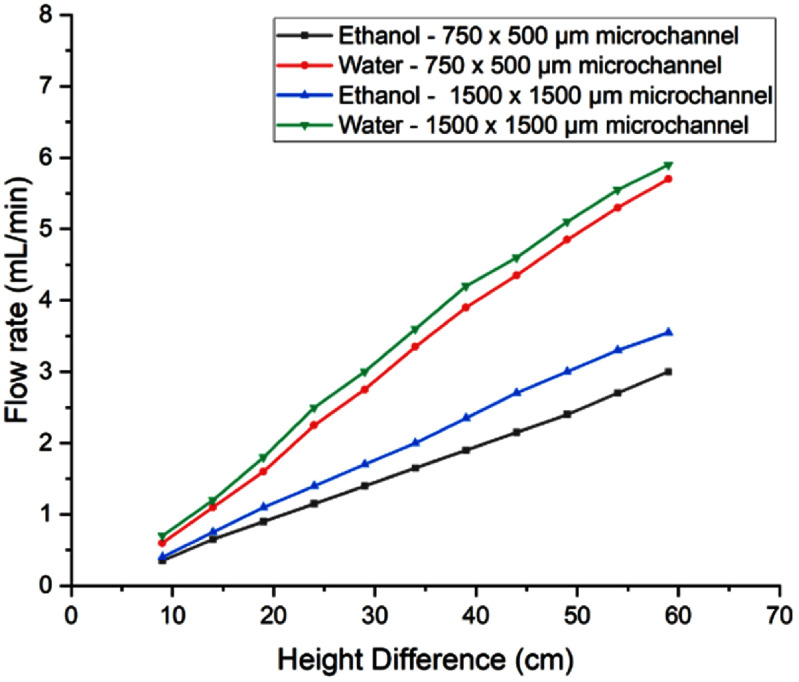
Relationship between height difference and gravity-driven flow rates of water and ethanol through microchannels with dimensions of 750 × 500 *µ*m and 1500 × 1500 *µ*m.

The effect of height difference on gravity-driven flow was evaluated by measuring water and ethanol flow rates through rectangular microchannels with a cross section of 750 × 500 *µ*m and 1500 × 1500 *µ*m, respectively. Figure 2 is a graph plotted the relationship between height difference and the measured flow rates of water and ethanol passed through different microchannel dimensions. A key observation is the difference in slope between water and ethanol lines. Owing to its higher viscosity, ethanol has smaller slopes, requiring greater height differences to achieve the same flow rates. For example, to reach 3 ml min^−1^ through a 1500 × 1500 *µ*m channel, water required a height difference of 32 cm, while ethanol required 49 cm. In addition, channel dimensions revealed a clear impact on the gravity-driven flow rates. In larger channels, wall frictional resistance is reduced, facilitating smoother liquid transport and requiring a lower height to achieve the same flow rate. This trend is illustrated in figure 2 graph, where for each liquid the narrower 750 × 500 *µ*m channel consistently gives lower flow rates than the larger 1500 × 1500 *µ*m channel under the same height difference. For instance, at a height difference of 50 cm, ethanol flowed through the narrower channel at 2.4 ml min^−1^, whereas its flow rate through the larger channel was 3 ml min^−1^. Accordingly, the nearly parallel lines for each liquid across different channel sizes indicate that viscosity predominantly determines the differences between ethanol and water, while channel geometry primarily influences the overall flow magnitude.

### 3.2. Characterization and optimization of sharp-tip mixing for the gravity-driven flow device

**Figure 3.**
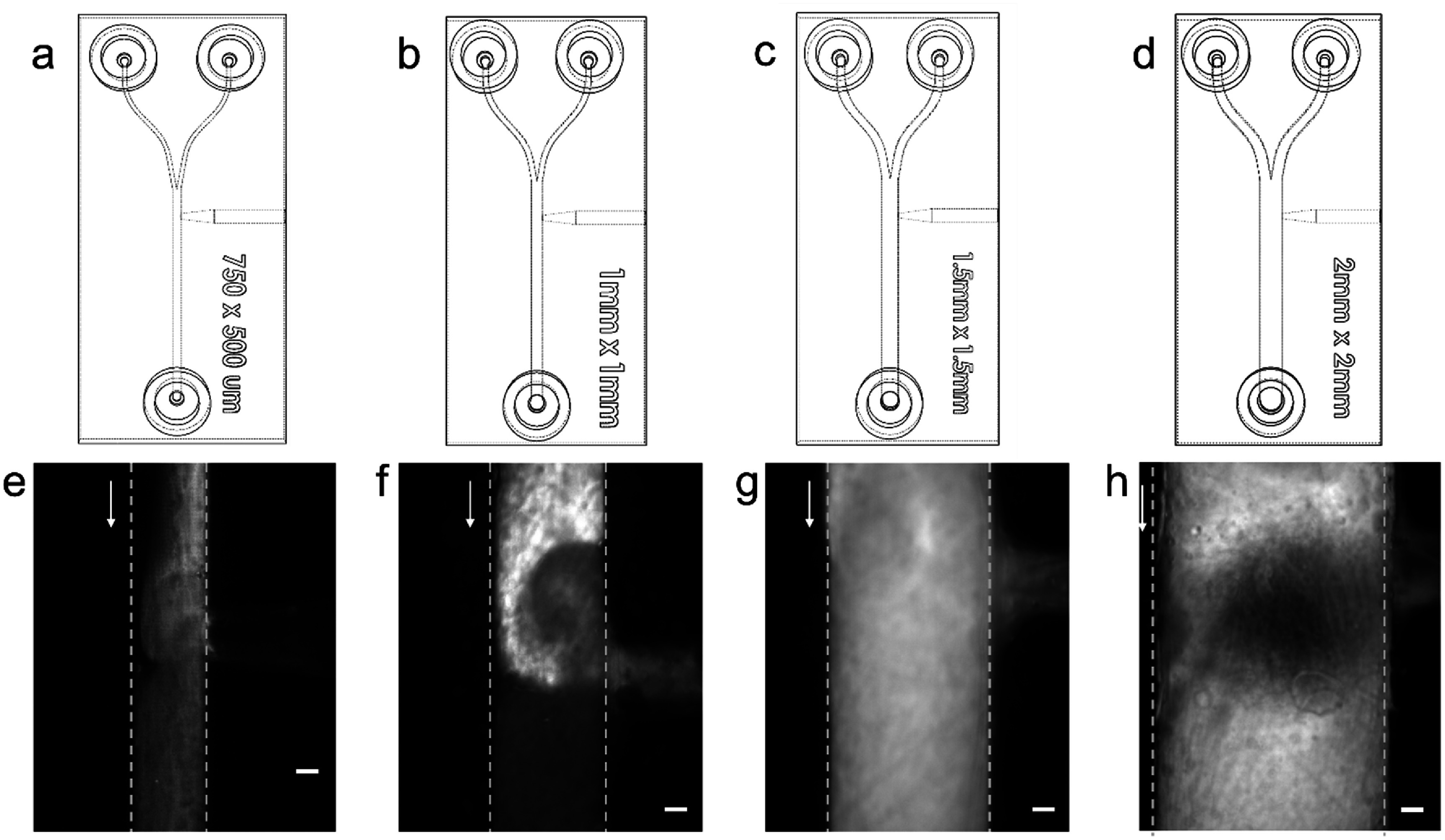
Schematic of the microfluidic chip showing rectangular main channels with dimensions of (a) 750 × 500 *µ*m, (b) 1000 × 1000 *µ*m, (c) 1500 × 1500 *µ*m, and (d) 2000 × 2000 *µ*m. Corresponding fluorescence microscopy images of mixing performance in sharp-tip microfluidic devices with channel dimensions of (e) 750 × 500 *µ*m, (f) 1000 × 1000 *µ*m, (g) 1500 × 1500 *µ*m, and (h) 2000 × 2000 *µ*m. Images were acquired under optimal mixing conditions at a total flow rate of 3 ml min^−1^. Arrows indicate flow direction and the scale bar is 200 *µ*m.

The present mixing method is enabled by the strong acoustic streaming induced by a vibrating sharp-tip capillary. As reported by Ovchinnikov *et al*, the strong acoustic streaming is caused by the geometrical focusing effect of the sharp edge structure [27]. As the fluid moves around a wedge, the streamlines must bend sharply and crowd together near the apex. As the tip moves back and forth due to vibration, no-slip shear produces strong vorticity inside the viscous layer. The Navier–Stokes equation contains the nonlinear convective term, which converts the large oscillatory gradients into a strong steady force. This strong steady force leads to the generation of a second order steady state fluid flow, acoustic streaming. The double vortices streaming pattern is further confirmed by previous numerical studies [27, 28]. To enable efficient LNP synthesis under gravity-driven flow, we optimized the sharp-tip mixing microfluidic device system to improve compatibility with gravity-driven flow at higher flow rates while maintaining effective mixing. We first studied the optimal cross section of the main channel to ensure stable flow and efficient mixing. Bigger channel cross section leads to lower resistance and higher throughput at the same height difference but presents challenge to achieve uniform mixing across the whole channel. In this regard, multiple microfluidic devices with different main channel geometries were designed and fabricated, as illustrated in figures 3(a)–(d). Subsequently, each design was then integrated into the gravity-driven flow setup and evaluated under fluorescence microscopy. We tested 4 different cross sections: 1) 750 × 500 *µ*m (w*h); 2) 1000 × 1000 *µ*m; 3) 1500 × 1500 *µ*m; 4) 2000 × 2000 *µ*m. Figures 3(e)–(h) show the corresponding fluorescence microscopy images of mixing performance at a total flow rate of 3 ml min^−1^. As indicated in figures 3(e)–(g), complete and uniform mixing was observed in devices with channel dimensions up to 1500 × 1500 *µ*m, indicating efficient mixing under gravity-driven flow at these cross sections. In contrast, the largest channel with dimensions of 2000 × 2000 *µ*m exhibited persistent air bubble formation and incomplete mixing, as shown in figure 3(h), likely due to insufficient interaction between the fluid streams and vibrating sharp-tip within the larger channel volume. Based on these observations, the 1500 × 1500 *µ*m channel cross section was selected as the optimal design, as it reduces hydrodynamic resistance compared to smaller channels while providing a balance between high throughput, stable gravity-driven flow, and efficient mixing required for synthesis.

**Figure 4.**
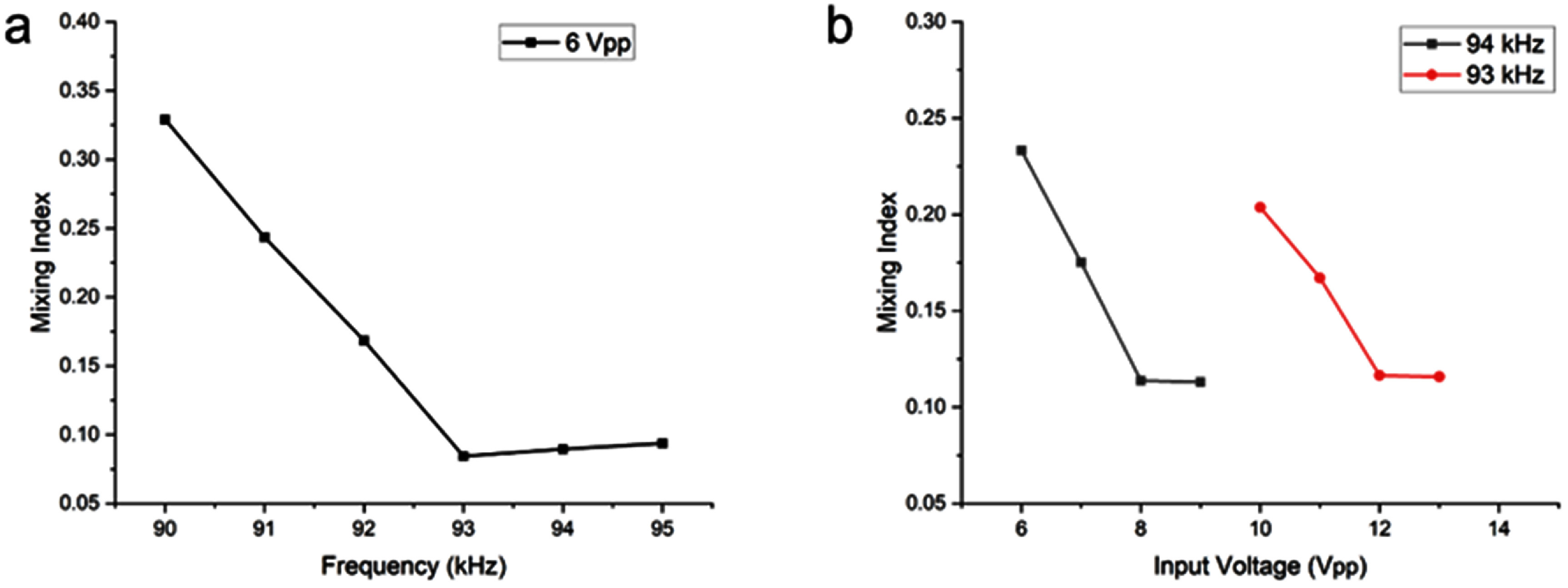
Mixing performance of the sharp-tip microfluidic device (a) at different frequencies under fixed 6 *V*_pp_ amplitude and 1 ml min^−1^ TFR, (b) at varying amplitudes for 93 and 94 kHz at 5 ml min^−1^ TFR.

Prior to LNP synthesis, the mixing performance of the sharp-tip microfluidic system and its dependency on actuation frequency and amplitude were studied using a 3D-printed chip with a 1500 × 1500 *µ*m microchannel and a short, graduated taper sharp-tip. Mixing efficiency in the sharp-tip device depends strongly on the actuation conditions, particularly the applied frequency and voltage amplitude, which determine the strength and structure of the acoustically induced vortices. Therefore, we investigated the frequency and power dependence of mixing performance under gravity-driven flow. In this regard, the effect of actuation frequency at a fixed amplitude was examined. Figure 4(a) shows the mixing index as a function of frequency at an amplitude of 6 *V*_pp_ and a total flow rate of 1 ml min^−1^. Among the tested frequencies, both 93 and 94 kHz produced the lowest mixing index values, indicating complete mixing. Typically, the lowest frequency is selected as the optimal one for subsequent amplitude evaluation. However, in this study, to further examine the influence of frequency at higher flow rates, both frequencies were evaluated over a range of voltage amplitudes. Figure 4(b) presents the mixing index values at varying amplitudes for 93 and 94 kHz at a TFR of 5 ml min^−1^. A clear frequency-dependent difference was observed in the minimum power required for efficient mixing at higher flow rates. Accordingly, at 94 kHz, complete mixing was achieved at an amplitude of 8 *V*_pp_, whereas at 93 kHz a higher amplitude of at least 12 *V*_pp_ was required to reach a comparable mixing index. Although both 94 kHz–8 *V*_pp_ and 93 kHz–12 *V*_pp_ actuation combinations ultimately produced efficient mixing with identical mixing index values and provided reliable performance for nanoparticle synthesis, the substantially lower voltage requirement at 94 kHz highlights the critical role of frequency selection in reducing power consumption while maintaining efficient mixing. These results demonstrate that frequency plays a key role in determining mixing outcome, particularly at high flow rates, where operation at an optimal frequency enables complete mixing at significantly lower input voltage.

### 3.3. LNPs synthesis and characterization

**Figure 5.**
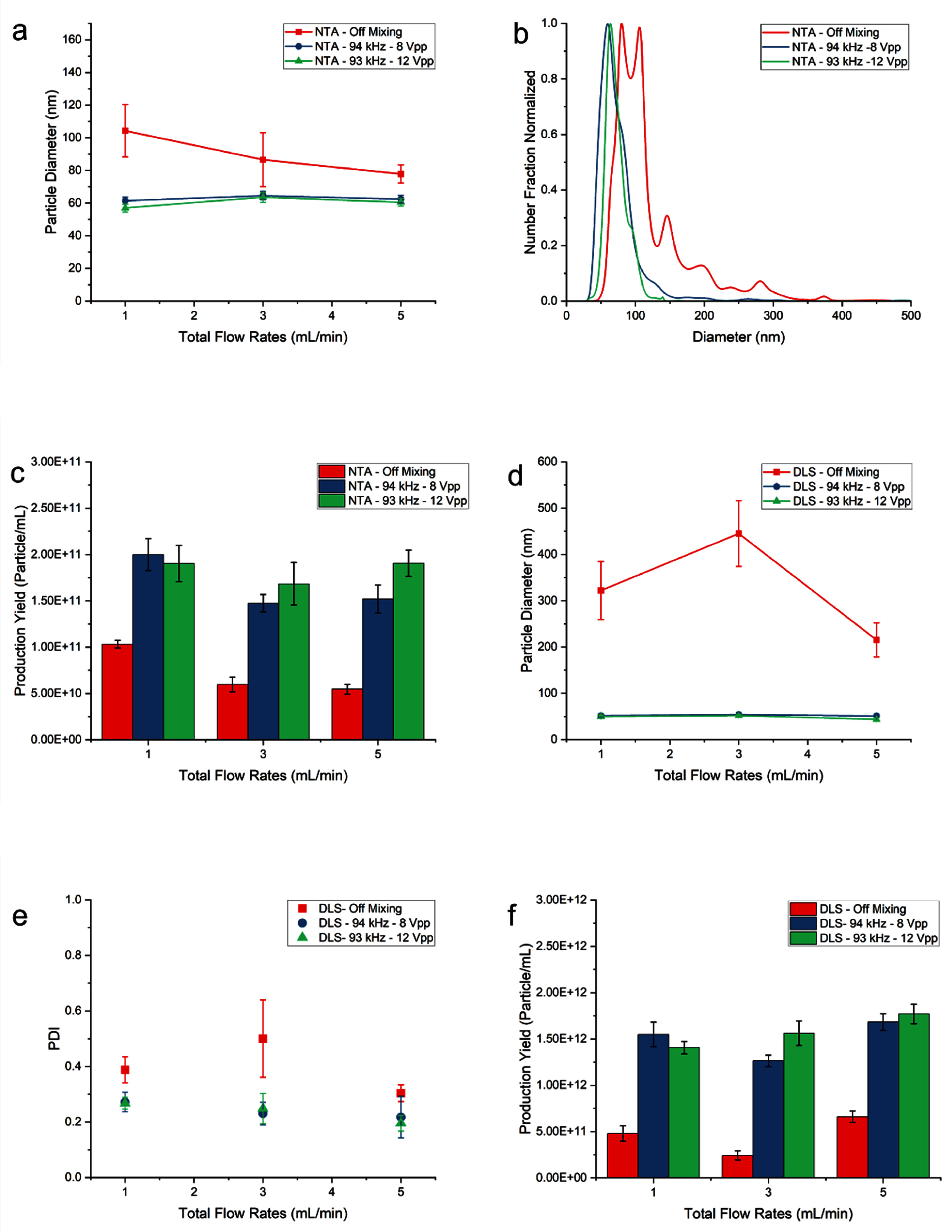
Characterization of POPC nanoparticles synthesized at TFRs of 1, 3, and 5 ml min^−1^ under gravity-driven flow via the sharp-tip mixing integrated with microfluidic chip with a 2.3 mm junction to side channel distance using mixing conditions of no mixing, 94 kHz–8 *V*_pp_, and 93 kHz–12 *V*_pp_. NTA results show (a) particle diameter, (b) size distribution at 5 ml min^−1^, and (c) production yield. DLS results show (d) particle diameter, (e) PDI, and (f) production yield. Data are presented as mean ± standard deviation from six independent experiments (*n* = 6).

With the optimal setup and operational parameter, POPC LNPs were then synthesized using the sharp-tip capillary mixing device under the gravity-driven flow. By adjusting the reservoir heights, nanoparticles were synthesized at three different TFRs of 1, 3, and 5 ml min^−1^, while the FRR was fixed at 1:3 of saline solution over ethanolic lipid solution. Additionally, to evaluate the impact of mixing condition, the synthesis process at each TFRs was repeated under three conditions: no mixing and mixing at two different actuation setups with frequency and amplitude combinations of 94 kHz–8 *V*_pp_ and 93 kHz—12 *V*_pp_, respectively. While both actuation setups demonstrated complete mixing, their input voltage and the acoustic streaming they created are different. At the initial step, based on the optimization of the sharp-tip mixing device for gravity-driven flow, the 3D-printed chip featuring 1500 × 1500 *µ*m microchannel integrated with the short, graduated taper was employed to synthesize POPC LNPs. The resulting LNPs were characterized using NTA to assess particle size, and production yield. Also, since NTA does not provide polydispersity index (PDI) value, dynamic light scattering (DLS) with a Zetasizer Pro instrument was employed to evaluate the uniformity of the product. Figures 5(a)–(c) illustrates the NTA characterization of nanoparticles produced with and without active mixing. As displayed in figure 5(a), nanoparticles synthesized without active mixing showed considerably larger sizes compared to those produced with active mixing. Statistical analysis (*t*-test, *n* = 6) also confirmed that both actuation conditions produced significantly smaller nanoparticles compared to the off-mixing condition at all tested flow rates (*p* < 0.01 to *p* < 0.001), while no statistically significant difference was observed between the two actuation conditions at 3 and 5 ml min^−1^ (*p* > 0.05). Although this difference between off-mixing and on-mixing decreased at higher TFRs, as the size of nanoparticles synthesized without mixing decreased with increasing TFRs. Specifically, at 5 ml min^−1^ without mixing, the average nanoparticle size reduced to 77.8 ± 5.6 nm, which was closer to the 62.5 ± 3.4 nm obtained under active mixing at 94 kHz–8 *V*_pp_. This reduction in the size of nanoparticles is attributed to the increased pressure at the converging junction under higher flow rates, which forces the two streams to meet more intensively and thereby enhances molecular diffusion between the solutions. Additionally, at higher TFRs, limited molecular diffusion along with reduced residence time in the channel restricts particle growth, thereby maintaining smaller nanoparticle sizes.

Although nanoparticles synthesized without mixing at 5 ml min^−1^ were smaller and closer in size to those obtained with active mixing, figure 5(b) shows that their size distribution remained highly polydisperse, indicating insufficient molecular diffusion to achieve uniform nanoparticle formation. In contrast, nanoparticles synthesized with active mixing using the sharp-tip capillary not only possessed smaller sizes but also demonstrated much narrower size distributions, indicating improved product uniformity and highlighting the effectiveness of active mixing in controlling both size and polydispersity. Also, the average sizes of nanoparticles generated at both actuation combinations were almost identical and showed no significant variation with increasing TFRs.

Furthermore, the estimated production yield of nanoparticles synthesized with and without active mixing was evaluated using NTA as shown in figure 5(c). In this regard, nanoparticles produced without active mixing showed a marked drop in yield, averaging 1.03 × 10^11^ ± 0.04 × 10^11^, 5.97 × 10^10^ ± 0.79 × 10^10^, and 5.46 × 10^10^ ± 0.54 × 10^10^ particles/ml at TFRs of 1, 3, and 5 ml min^−1^, respectively. In contrast, active mixing increased the yield by nearly an order of magnitude, with differences of about 1.0 × 10^11^ particles/ml between conditions. For example, at 3 ml min^−1^, active mixing with the 93 kHz–12 *V*_pp_ actuation combination produced 1.68 × 10^11^ ± 0.23 × 10^11^, particles/ml, compared to 5.97 × 10^10^ ± 0.79 × 10^10^ particles/ml for nanoparticles synthesized without mixing. Though, active mixing at both 94 kHz–8 *V*_pp_ and 93 kHz–12 *V*_pp_ actuation combinations resulted in very close yields at 1 and 3 ml min^−1^, with values of 2.0 × 10^11^ ± 0.17 × 10^11^ and 1.90 × 10^11^ ± 0.20 × 10^11^ particles/ml at 1 ml min^−1^, and 1.47 × 10^11^ ± 0.09 × 10^11^ and 1.68 × 10^11^ ± 0.23 × 10^11^ particles/ml at 3 ml min^−1^, respectively. However, at 5 ml min^−1^, 93 kHz–12 *V*_pp_ actuation achieved a slightly higher yield of 1.91 × 10^11^ ± 0.14 × 10^11^ particles/ml compared to 1.52 × 10^11^ ± 0.15 × 10^11^ particles/ml for 94 kHz–8 *V*_pp_, confirming that higher input voltage with enhanced vortex strength improves nanoparticle production efficiency at elevated flow rates. To confirm these findings, statistical analysis (*t*-test, *n* = 6) was conducted, demonstrating that both actuation conditions significantly enhanced production yield compared to the off-mixing condition at all flow rates (*p* < 0.0001). Also, no statistically significant difference was observed between the two actuation conditions at 1 and 3 ml min^−1^ (*p* > 0.05), whereas a difference was detected at 5 ml min^−1^ (*p* < 0.01).

To further validate the results and confirm their uniformity by analyzing PDI value, all samples were also characterized by DLS. The size, PDI, and estimated concentration of the products are presented in figures 5(d)–(f). Consistent with the NTA results, nanoparticles synthesized without mixing were considerably larger than those produced with active mixing, as shown in figure 5(d). However, the relationship between nanoparticle size and TFR cannot be reliably evaluated here, since in polydisperse samples DLS has substantially lower sensitivity to smaller sized nanoparticles compared to NTA [29]. In contrast, under active mixing, the nanoparticles remained significantly smaller across all TFRs, highlighting the effectiveness of sharp-tip mixing in controlling particle size. The average sizes of nanoparticles synthesized with active mixing at 94 kHz–8 *V*_pp_ were 51.5 ± 3.5, 53.9 ± 4.3, and 50.9 ± 3.9 nm for TFRs of 1, 3, and 5 ml min^−1^, respectively. Similarly, nanoparticles produced using 93 kHz–12 *V*_pp_ actuation showed average sizes of 49.8 ± 3.4, 52.0 ± 2.5, and 43.3 ± 3.0 nm at the same TFRs, which were very close to those obtained with 94 kHz–8 *V*_pp_ at 1 and 3 ml min^−1^. At 5 ml min^−1^, however, the nanoparticles synthesized at 93 kHz–12 *V*_pp_ were smaller than those at 94 kHz–8 *V*_pp_, confirming that higher input voltage and stronger vortex generation can play a role at higher flow rates in reducing nanoparticle size.

Polydispersity is a critical quality parameter in microfluidic nanoparticle synthesis, as it directly reflects the uniformity and consistency of particle size distribution. The PDI of nanoparticles synthesized using the sharp-tip mixing microfluidic device under gravity-driven flow was characterized by DLS. Typically, the PDI value below 0.3 is considered as monodisperse with uniform size distribution of nanoparticles, acceptable for biomedical applications [30]. According to figure 5(e), nanoparticles synthesized in the absence of active mixing revealed PDI values above 0.3, indicating polydisperse products, whereas those synthesized with active mixing consistently showed uniform distribution with PDI values below 0.3 across all actuation combinations and TFRs. In particular, for the 93 kHz–12 *V*_pp_ actuation combination, the PDI values were 0.26 ± 0.02, 0.24 ± 0.05, and 0.19 ± 0.02 at TFRs of 1, 3, and 5 ml min^−1^, respectively, while for the 94 kHz–8 *V*_pp_ actuation combination, the corresponding values were 0.27 ± 0.03, 0.23 ± 0.04, and 0.21 ± 0.02. *T*-test statistical analysis (*n* = 6) indicated that both actuation conditions significantly decreased PDI relative to the no-mixing condition at all flow rates (*p* < 0.01 to *p* < 0.0001), with no significant difference between actuation conditions across all TFRs (*p* > 0.05). Moreover, under active mixing conditions, the PDI values at all TFRs overlapped between the two utilized actuation combinations, while showing a falling trend with growing TFRs, highlighting improved uniformity at higher flow rates.

Lastly, the production yield of nanoparticles was characterized using DLS and is shown in figure 5(f). Similar to the NTA results, not only was the yield with active mixing significantly higher than that without mixing, but the estimated yields between the two actuation combinations were also very close to each other. These results highlight the unique contribution of the present vibrating sharp-tip mixing device to LNP synthesis. It strikes a fine balance between the mixing efficiency and channel dimension.

In addition, we studied influence of the distance between the side channel and the device converging junction on the quality of the LNPs. We compared two distances, 3.3 mm and 2.3 mm, respectively. In the 3.3 mm design, the vortices generated by the vibrating sharp-tip cannot fully cover the converging junction, whereas the 2.3 mm design allows the vortices to fully cover the converging junction (figure S1).

As illustrated in figure S2, the 3.3 mm design led to the two inlet streams to interact via molecular diffusion over a longer distance before entering the vortex region where active mixing occurs. Characterization of LNPs synthesized using this design is presented in figure S3. The results showed a strong dependence on side channel positioning, with nanoparticles exhibiting high polydispersity, as indicated by PDI values exceeding 0.3 across most TFRs, even in the presence of active mixing. In addition, lower production yields were observed compared to the optimized device with the shorter side channel distance of 2.3 mm. These findings demonstrate that when the acoustic vortex does not extend to the converging junction, premature diffusion can initiate uncontrolled nanoparticle formation upstream of the active mixing zone, thereby reducing control over size uniformity. This study revealed that factors beyond mixing strength, particularly the spatial location of mixing and nanoparticle formation within the microchannel, play a critical role in determining product uniformity and quality.

We also evaluated the stability of the LNPs synthesized using the gravity flow-based method. LNPs 2 h after production and after 72 h of storage under two different conditions: (i) storage in the original nanoparticle suspension collected directly from the microfluidic device and (ii) storage after dilution in deionized water were studied. The results obtained from both NTA and DLS showed consistent trends, demonstrating that LNPs stored in their original suspension maintained their particle size and PDI with only minimal changes over the 72 h storage period (figure S4). This behavior is attributed to the electrostatic interactions and electrical double layer surrounding the particles, which provide colloidal stability and prevent particle aggregation. In contrast, LNPs diluted in deionized water exhibited an increase in particle size after 72 h. The reduced ionic environment weakens the stabilizing interactions between particles, making aggregation more likely. Furthermore, Ostwald ripening may also contribute to this behavior, whereby smaller particles gradually falling apart and redeposit onto larger particles, resulting in the growth of larger nanoparticles over time.

In addition, we also compared LNPs synthesized via sharp-tip using gravity-driven flow with the NanoAssemblr using syringe-pump at TFRs of 1 ml min^−1^ and FRR of 1:3. As shown in figure S5, the size, size distribution, and yield are highly comparable between the two methods, indicating that the gravity flow-based method does not sacrifice the quality of LNPs compared with a syringe pump-based commercial device.

Finally, we tested the encapsulation efficiency of the present method for ssDNA molecules. Here we adopted a widely used recipe for LNP encapsulation, 50: 10: 38.5: 1.5 for DOTAP, DSPC, cholesterol, and DMG-PEG 2000. The encapsulation cargo is a 20 mer ssDNA labeled with FAM for encapsulation efficiency characterization. After the synthesis experiment, the unencapsulated DNA was removed using an ultrafiltration centrifugal concentrator (100 K MWCO). The DNA-LNP sample was then lysed using 1% Triton X-100 before measuring fluorescence emission at 515 nm using a fluorometer. Based on the theoretical total concentration of ssDNA, encapsulation efficiency was calculated to be 55%±4% for 3 replicates, indicating the successful encapsulation of ssDNA using the present methods. Further improvement in encapsulation efficiency is expected after optimizing the recipe and FRR of the present method.

## 4. Conclusion

In this work, we demonstrated a gravity-driven sharp-tip microfluidic platform capable of synthesizing LNPs with high throughput and narrow size distribution. By replacing conventional active pumps with gravity-driven flow, the system significantly reduces the resources needed while maintaining stable and controllable flow conditions at total flow rates up to 5 ml min^−1^. Optimization of microchannel cross section identified a 1500 × 1500 *µ*m geometry as an optimal balance between low hydrodynamic resistance, flow stability, and effective acoustic mixing. The presence of active sharp-tip mixing markedly improved nanoparticle synthesis by enhancing yield and uniformity, whereas the absence of mixing led to uncontrolled nucleation, broader size distributions, and reduced reproducibility. Under optimized gravity-driven conditions with active mixing, the platform demonstrated consistently production of high-quality LNPs with sizes in the 50–60 nm range and PDI values below 0.3, demonstrating monodisperse and uniform products suitable for biomedical applications.

While present work demonstrates the potential of gravity driven flow for LNP manufacturing, the throughput needs to be further scaled up for practical applications. One straightforward strategy is via parallelization. Parallelization is highly compatible with the present method due to the low cost and good energy efficiency of each individual unit (∼1 J mg^−1^ of lipid production). The fluidic device is 3D printed using low-cost bench top 3D printer (<$400), and the sharp-tip device cost <$2 due to the simple components it needs. For each sharp tip, the energy consumption is ∼80 mW. Therefore, there is no major obstacle to the parallelization of the present method. However, to achieve full automation, a simple and low-cost sample delivery and replenishment system is needed.

It should also be noted that there are still inherent challenges in utilizing gravity for LNP manufacturing, including flow rate consistency, potential disturbances due to air bubbles and pressure fluctuation, and the variations in viscosity for different LNP recipes. The present method offers more tolerance to flow fluctuations and variations. Unlike hydrodynamic-based mixing, the mixing performance of the present method is insensitive to the flow rate variations, providing that the flow rate is below the maximum allowed flow rate. Therefore, despite some flow rate variations the mixing performance is likely to be maintained. In addition, process automation strategies, such as continuous or feedback-controlled replenishment of the reservoir, can be adopted to maintain a constant liquid head.

Overall, the platform presented here demonstrates a unique alternative to pump-driven microfluidic systems and offers a general design framework for sustainable, high-throughput nanomanufacturing.

## Data Availability

Additional data that support the findings of this work are available from the corresponding author upon reasonable request. The data cannot be made publicly available upon publication because no suitable repository exists for hosting data in this field of study. Supporting Information available at: https://doi.org/10.1088/1758-5090/ae94b4/data1.
